# Toward a Reasoned Classification of Diseases Using Physico-Chemical Based Phenotypes

**DOI:** 10.3389/fphys.2018.00094

**Published:** 2018-02-28

**Authors:** Laurent Schwartz, Olivier Lafitte, Jorgelindo da Veiga Moreira

**Affiliations:** ^1^Assistance Publique des Hôpitaux de Paris, Paris, France; ^2^LAGA, UMR 7539, Paris 13 University, Sorbonne Paris Cité, Villetaneuse, France; ^3^Interfaces, Paris-Saclay University, Palaiseau, France

**Keywords:** disease classification, physico-chemical forces, cell membrane, metabolism, mitochondria

## Abstract

**Background:** Diseases and health conditions have been classified according to anatomical site, etiological, and clinical criteria. Physico-chemical mechanisms underlying the biology of diseases, such as the flow of energy through cells and tissues, have been often overlooked in classification systems.

**Objective:** We propose a conceptual framework toward the development of an energy-oriented classification of diseases, based on the principles of physical chemistry.

**Methods:** A review of literature on the physical chemistry of biological interactions in a number of diseases is traced from the point of view of the fluid and solid mechanics, electricity, and chemistry.

**Results:** We found consistent evidence in literature of decreased and/or increased physical and chemical forces intertwined with biological processes of numerous diseases, which allowed the identification of mechanical, electric and chemical phenotypes of diseases.

**Discussion:** Biological mechanisms of diseases need to be evaluated and integrated into more comprehensive theories that should account with principles of physics and chemistry. A hypothetical model is proposed relating the natural history of diseases to mechanical stress, electric field, and chemical equilibria (ATP) changes. The present perspective toward an innovative disease classification may improve drug-repurposing strategies in the future.

## Introduction

Diseases have been historically classified according to their nature, anatomical site, association to an external cause, and the cause of death (Moriyama et al., 2011). The International Classification of Diseases (ICD) provides a health care classification that has been based on these criteria. In its 11th revision, the ICD-11 proposes different dimensions for a given disorder as a function of its topography, temporal properties (acute, chronic), severity, clinical phenotypes (signs and symptoms), and etiology (Jiang et al., 2013). Classification of diseases has excluded the influence of physical and chemical forces governing transformation of energy in both matter and living systems. Energy takes many forms in biological systems. At the cellular level, biological functions are primarily regarded as being influenced by electric, magnetic, light, mechanical, heat, and chemical energies. Heat is cyclically exchanged between cells and environment. Plants, algae, and cyanobacteria absorb light energy via photosynthesis, whereas nuclear forces have only a negligible impact on living systems. Excluding infections, most diseases have unknown etiology, and more or less unclear pathophysiology. In the late 1850s, Louis Pasteur and Robert Koch, put forward the germ theory, according to which diseases are caused by infectious microbes, impairing functioning of structures of different organ systems (Pasteur, 1881). The concept of etiology, or disease's specific causation, was crystallized by the germ theory (Carter, 1980). The physiologist Claude Bernard, Pasteur's contemporary and friend, argued instead for importance of the balance in body internal environment—what he called le *milieu intérieur* (Bernard, 2013). The other points in his argument are that the physical and chemical sciences provide the foundation for physiology and that biology depends on recognizing that the processes of life are mechanistically determined by physico-chemical forces (Bernard, 1865). Here, we put forward a conceptual framework outlining integrative approaches to classify diseases based on physico-chemical-based phenotypes. These dimensions comprise the same laws that govern inorganic and organic matter.

## Physico-chemical fundamental underpinnings of physiological processes

### Chemistry governs cellular metabolism

Metabolism is a physico-chemical process which involves the chemical conversion of energy into biological work (Lehninger, 1971). Molecules are absorbed through pores in the membrane and they react to break down molecules to generate energy used in heat formation, which when dissipated maintains body temperature constant and in synthesis of nucleic acids, proteins, and lipids. In a thermodynamic sense, cells can be viewed essentially as an isothermal combustion engine engaged in a Carnot cycle, performing work and generating heat, thus requiring a constant supply of energy-giving molecules like glucose (Fermi, 1956; Lehninger, 1971). Cell metabolism is the sum of all the chemical reactions and dynamic exchanges between a cell and its microenvironment. Utilization of free energy from molecular bond rearrangement of nutrients powers biological processes in every biological organism.

Eukaryote cells, exhibit two opposite metabolisms: catabolic reactions, leading to the breakdown of macromolecules for energetic use and anabolic reactions, which consists of synthesis of biomass. Cells convert energy by means of an electron-proton transfer process to produce ATP. The energy of electron flow is stored under the form of chemical free energy of ATP, which is then used to execute the mechanical, osmotic, and biosynthetic work of cells (Lehninger, 1965, 1971). Metabolic networks continue to generate the requisite amount of energy after removal of certain reactions, characterizing stability and resilience in the face of endogenous and exogenous perturbation (Demetrius, 2013). The standard energy of ATP hydrolysis remains within a narrow range among cells with widely varying membrane potential and mechanisms of energy production (Seyfried and Shelton, 2010; Lane and Martin, 2012). Oxidative phosphorylation (OxPhos) provides about 88% of the total energy and substrate phosphorylation (mainly glycolysis) contributes the remaining 12%. In OxPhos, which occurs within mitochondria, electrical charges are transferred to oxygen via redox reactions and protons are pumped from the matrix across the mitochondrial inner membrane. ATP is synthesized when protons return to the mitochondrial matrix down their electrochemical gradient. The rate of energy production in OxPhos is determined by the conductance of the bio-membrane and the electromotive potential across the membrane (Nicholls et al., 2002). Energy production in glycolysis, however, is independent of electrical gradients. Now, the rate of energy production is determined by the activity of the glycolytic enzymes of the cytoplasm, without exchanging charges with dielectric membranes (Demetrius et al., 2010).

Cell differentiation and proliferation are at least in part controlled by the intracellular pH. Differentiated cells have a lower pH than proliferating cells (Lee et al., 2003). Pouyssegur's group showed that cells cannot proliferate when the intracellular pH is below 7.2 (Sardet et al., 1989). pH change inside cells can be explained by several phenomena, such as the sodium/proton transmembrane exchanger (Moolenaar et al., 1981; Boron, 2004). The intracellular pH plays a key role in determining the way cells allocate energy, especially driving the switch between OxPhos and glycolysis (da Veiga Moreira et al., 2015). At acidic pH, cytoplasmic activity of ATPase is inhibited and mitochondrial respiration is optimal, implicating increased ATP concentrations. On the other hand, when cytoplasmic pH is alkaline, ATP concentration falls, probably due to impaired mitochondrial respiration and increased ATPase activity (Christen et al., 1983). Moreover, it has been demonstrated that intracellular pH drives protein synthesis and DNA replication (Busa et al., 1982; Busa and Crowe, 1983; Hand and Carpenter, 1986). Intracellular acidic pH is followed by global histone deacetylation, leading to chromatin compaction, the phenotype of a dormant cell, like a myocyte or a neuron. Conversely, intracellular pH increase toward alkalinisation is reported to favor acetylation of histone, leading to chromatin decompaction and DNA replication (McBrian et al., 2013; Kurdistani, 2014). These phenomena occur after resting cells are committed to proliferate, such as in cancer. All such chemical equilibria contribute to the chemical driving forces distribution at play in the body.

### Mechanical forces underlie cell biology

The influence of mechanical energy of living organisms is omnipresent. Cells are continuously subjected to stretching, compression, and shear forces that influence cell division, gene expression, cell migration, morphogenesis, cell adhesion, fluid homeostasis, ion channel gating, and vesicular transport (Hamill and Martinac, 2001; Kim et al., 2009; Eyckmans et al., 2011). The seminal work of D'Arcy Thompson demonstrated that mechanical forces play a key role in plant and animal morphogenesis (Thompson, 1942). These physical forces displace the relative locations of molecules within cells and tissues, which give rise to viscoelastic deformation of membranes and cytoskeletal and extracellular matrices (Eyckmans et al., 2011). We already have an intuitive understanding of the distribution of mechanical forces when we consider pressure, which depends not only on environmental and endogenous loads (pressure exerted by cavities and blood) but also on intrinsic mechanical factors of organs, such as shape, architecture, and mechanical properties of tissues (Brinckmann et al., 2002; Jacobs et al., 2012; Levy Nogueira et al., 2016).

Fluid mechanics can influence cell function via osmotic pressure. This form of pressure is exerted when water is transported across a semi-permeable membrane, a membrane allowing only water molecules but none of solute molecules to pass through (DeDuve, 1991). Oncotic pressure is a form of osmotic pressure exerted by proteins, notably albumin, in blood plasma that usually tends to pull water from interstitial, lymphatic and cerebrospinal fluids, into the circulatory system. Enhanced anabolism results into production of dissolved biomass products, hence in increased osmotic and oncotic pressures. It is the opposing force to hydrostatic pressure, generated by the weight of a liquid in presence of gravity. In blood circulation, hydraulic pressure changes with body posture. The hydraulic pressure is due to the external force acting on a surface of a liquid; in blood circulation, the origin of this force is the heart and blood vessels. When a liquid flows, a dynamic pressure is produced in the flow direction and the total pressure in this liquid is the sum of hydrostatic and dynamic pressures. All these forces contribute to the mechanical driving forces distribution at play in the body.

### Electrical forces drive cell membrane functions

Local electric fields within cells result mostly from the distributions of charged particles, such as ions Na^+^, K^+^, Ca^2+^, and Cl^−^ across phospholipid bilayer membranes by the opening and closing of channels. Such distributions result from diffusion and electrostatic forces generated by ion gradients and electrochemical potentials. The first cell studied from the point of view of the electricity was the neuron. The flow of electrical currents through an axon was firstly described by the cable theory, developed in the nineteenth century by Lord Kelvin to explain the flow of electricity in submarine cables. Cole, Goldman, Hodgkin and Katz adapted cable theory in the 1920–40s, considering resistances and capacitances of cells membranes and the properties of electrolytes that surround it (Goldman, 1943; Hodgkin and Huxley, 1952). Later, cable theory, Hodgkin and Huxley model and its modified versions took into account the influence of ion channels and ionic dynamics to study the electrical conduction and excitability of dendrites and neural networks (Butera et al., 1999; Stuart et al., 2016). More recently, synaptic currents have been more accurately described at nanoscale dimensions using the Poisson–Nernst–Planck equation and electro-diffusion modeling (Holcman and Yuste, 2015). Electrical currents propagate along the axon in neuronal networks but also it plays an important role in coordinating the contraction of the heart. Cardiac electrical potentials are generated by the sinoatrial node, the natural pacemaker of the heart and propagate from atria to ventricles via the atrioventricular node. Cardiac and skeletal muscle cell are excitable fiber conductors like neurons. In these cells, action potentials are triggered by arrival of synaptic currents at the neuromuscular junction. Electrical current plays also a key role in cell growth regulation and organogenesis (Pethig and Kell, 1987; Kubota et al., 1995; Thakral et al., 2013). All such electrical interactions contribute to the electric fields distribution at play in the body.

## Physico-chemical-based phenotypes of diseases

### Diseases exhibiting changes in metabolic rates: chemical phenotypes

Hypermetabolism, an increase in metabolic rate, is a hallmark of sustained pathophysiological stress response observed in fever, burn injury, severe trauma, and systemic inflammatory reaction in critically ill patients (Frankenfield et al., 1997; Porter et al., 2014). The initial description of the effects thyroid hormones on metabolic rate has made more than 100 years ago (Magnus-Levy, 1895). Thyroxin and triiodothyronin hormones, as well as adrenergic drugs, like epinephrine, and amphetamines increase metabolic yield (Nahorski and Rogers, 1973; Fisher et al., 1998; Ratheiser et al., 1998). Inversely, there are multiple mechanisms for decreased metabolic energy yield. The most common is probable age, a key risk factor for most diseases. Decreased metabolic rate may be due to lack of nutrients, such as in malnutrition, ischemia or anemia; or associated to some endocrine diseases like type 1 diabetes and hypothyroidism (Charlton and Nair, 1998; Singhal et al., 2002; Emery, 2005; McAninch and Bianco, 2016).

As stated by Otto Warburg almost 90 years ago, cancer is the simple consequence of altered metabolism (Warburg, 1956). In cancer cells, there is an increased uptake of glucose to compensate for the decrease energy yield, itself a consequence of a decreased mitochondrial activity. The pyruvate cannot be degraded via the Krebs cycle because the number of mitochondria is reduced (Levine and Puzio-Kuter, 2010; Schwartz et al., 2010; Israël and Schwartz, 2011; Porporato et al., 2011; Abolhassani et al., 2012). The mitochondrial defect results in decreased CO2 synthesis and alkalinization of the cytoplasm. A consequence of decreased mitochondrial activity is activation of the Pentose Pathway and tumor growth (Israël and Schwartz, 2011). In cancer, metabolic fluxes are diverted toward the pentose phosphate shunt rather than OxPhos, due to Warburg effect. Activation of this pathway results in synthesis of DNA and RNA (Levine and Puzio-Kuter, 2010; Schwartz et al., 2010; Israël and Schwartz, 2011; Porporato et al., 2011; Abolhassani et al., 2012). In cancer cells, the fuel is glucose and mitochondria do not secrete enough CO2 to acidify the cytoplasm. Therefore, the cytoplasm of the cancer cell is alkaline.

Mitochondrial dysfunction has been reported in numerous brain diseases—to name a few: Alzheimer's disease (AD) (Moreira et al., 2007), Parkinson's disease (Narendra et al., 2009), Huntington disease (Beal, 2005), bipolar disorders and schizophrenia (Clay et al., 2011). In AD, mitochondrial instability and dysfunction appear in a distinct population of neurons (Hirai et al., 2001). In order to sustain their energy needs, these neurons will disproportionally up-regulate OxPhos and consume more fuel substrates (lactate) (Demetrius and Driver, 2013). More vulnerable neurons, such as those from entorhinal cortex and hippocampus, could be damaged in consequence of glucose and lactate shortage. Circumscribed hypometabolism over these areas can be visualized in early stages of the disease of the brain [^18^F]fluorodeoxyglucose PET (FDG-PET) (Yakushev et al., 2011). This mode of neuroenergetic reprogramming is called the inverse Warburg effect (Demetrius et al., 2014). Inversely to cancer, the cytoplasm of brain cell in AD patients has an acidic pH in consequence of accumulation of lactic acid (Fang et al., 2010; Demetrius and Driver, 2013). Alkaline cytoplasm is strongly mitogenic, while acidic pH results in cell death (Zetterberg and Engström, 1981; Gottlieb et al., 1996).

### Diseases exhibiting changes in mechanical forces: mechanical phenotypes

During an acute inflammatory reaction, extravasation of plasma proteins occurs from the intravascular to the interstitial space (Table 1). For diagnostic proposes, clinicians currently identify high concentrations of protein in inflammatory fluids, such as in pleural effusion or pericarditis. This high protein content results in increased osmotic and colloid pressure. We have previously shown both *in vivo* and *in vitro* that hyperosmolarity can induce proinflammatory cytokine responses in epithelial cells (Abolhassani et al., 2008; Schwartz et al., 2008, 2009). Our group and others recently demonstrated that inflammation results from increased interstitial pressure (Grimble, 2003; Abolhassani et al., 2008; Schwartz et al., 2008, 2009). Osmotic forces have been linked to conditions like Crohn's disease, or ulcerative colitis (Schilli et al., 1982), ascites (Runyon, 1994), pericarditis (Szturmowicz et al., 1997), atherosclerosis (Blake and Ridker, 2002), arthritis (Sipe, 1995), pneumonia (Montón and Torres, 1998), and glaucoma (Flammer et al., 2002). The increased interstitial pressure may be responsible for common features of fibrosis and cancer. Increased fluid pressure is known to induce collagen deposition and modulate cell proliferation either by cell death or by cell multiplication (Schwartz et al., 2002). Cancer invades preferentially soft tissues such as glands or muscle rather than fascia or bone (Schwartz, 2004). Changes in physical constraints explain the stellar dendritic shape of cancer, enabling cells to escape physical constraints from their neighbors (Schwartz et al., 2002; Fleury and Schwartz, 2003). This functional polarity is most often lost during carcinogenesis (Locke, 1998).

**Table 1:** Mechanical phenotypes of diseases.

**Mechanical phenotype system**	**Decreased (insufficient)**	**Increased (accumulation of)**	**Tensile, compressive shear forces (Solid mechanics)**	**Hydrostatic hydrodynamic, osmotic and oncotic forces (Fluid mechanics)**
Osteomuscular	Osteopenia, osteoporosis (Bloomfield et al., 2016)^**^ Muscle atrophy (Bloomfield et al., 2016) - Due to long-duration bed rest, microgravity, limb paralysis or insufficient physical activity	Bone fracture (Mirzaali et al., 2015) Muscle and tendon rupture (Neviaser et al., 2012) Disk herniation (Gregory and Callaghan, 2010) Osteoarthritis (Silver and Bradica, 2002; Abramson and Attur, 2009; Vincent et al., 2012; Visser et al., 2014)	Silver and Bradica, 2002; Gregory and Callaghan, 2010; Neviaser et al., 2012; Vincent et al., 2012; Visser et al., 2014; Mirzaali et al., 2015; Bloomfield et al., 2016	Abramson and Attur, 2009
Cardiovascular	Orthostatic hypotension (Eiken et al., 2008) Hypovolemia, hypoperfusion (Kreimeier, 2000) Hypoxia, ischemia (Rumsey et al., 1994) Space Obstructive Syndrome (Wiener, 2012)	Arterial hypertension (Safar et al., 2003) Aneurysm and arterial dissection (Numata et al., 2016) Atherosclerosis (Anwar et al., 2012) Plaque rupture (De Korte et al., 2016) Cardiomyopathy (Modesto and Sengupta, 2014)	Kreimeier, 2000; Eiken et al., 2008; Anwar et al., 2012; Wiener, 2012; Modesto and Sengupta, 2014	Rumsey et al., 1994; Kreimeier, 2000; Safar et al., 2003; Eiken et al., 2008 Anwar et al., 2012; Modesto and Sengupta, 2014; De Korte et al., 2016; Numata et al., 2016
Respiratory	Hypoxic respiratory failure (Henderson and Sheel, 2012) High-altitude pulmonary edema (Swenson et al., 2002) Chronic Mountain Sickness (Villafuerte and Corante, 2016) Restrictive syndrome due to neuromuscular disorders (Celli, 2002)	Pulmonary hypertension (Puwanant et al., 2010) Pulmonary congestion (Picano and Pellikka, 2016) Chronic obstructive pulmonary disease (Bidan et al., 2015) Surfactant dysfunction (Douville et al., 2011)	Puwanant et al., 2010; Henderson and Sheel, 2012; Bidan et al., 2015; Villafuerte and Corante, 2016 Douville et al., 2011	Celli, 2002; Swenson et al., 2002; Puwanant et al., 2010; Douville et al., 2011; Henderson and Sheel, 2012; Bidan et al., 2015; Picano and Pellikka, 2016; Villafuerte and Corante, 2016
Digestive	Gastroesophageal reflux disease (Diamant, 2006; Pandolfino et al., 2010) Hiatus hernia (Diamant, 2006; Pandolfino et al., 2010)	Cirrhosis (Schwartz, 2014) Portal hypertension (Zhang et al., 2008)	Diamant, 2006; Zhang et al., 2008; Pandolfino et al., 2010	Zhang et al., 2008; Schwartz, 2014
Nervous	Spontaneous intracranial hypotension (Hasiloglu et al., 2012) High-altitude cerebral edema (Imray, 2016) High-altitude and microgravity headache (Wilson et al., 2011) Post-lumbar puncture headache and brain herniation (Kongstad and Grände, 1999)	Adult chronic hydrocephalus (Orešković and Klarica, 2011) Chronic traumatic encephalopathy (McKee et al., 2013; Stein et al., 2014) Alzheimer's diseases (Levy Nogueira et al., 2015a,b) Parkinson's disease (Goldman et al., 2006) Amyotrophic lateral sclerosis (Pupillo et al., 2012) Noise-induced hearing loss (Sun et al., 2015)	Goldman et al., 2006; Wilson et al., 2011; Levy Nogueira et al., 2015a,b Kongstad and Grände, 1999; McKee et al., 2013; Stein et al., 2014 Pupillo et al., 2012; Sun et al., 2015	Kongstad and Grände, 1999; Wilson et al., 2011; Hasiloglu et al., 2012; Imray, 2016 Orešković and Klarica, 2011; Sun et al., 2015
Systemic conditions	Fluid shifts ^*^ (Bloomfield et al., 2016; Johnson and Luks, 2016) Soft tissue changes due to microgravity (Avula, 1994)	Fluid shifts^*^ (Bloomfield et al., 2016; Johnson and Luks, 2016) Soft tissue damage due to traumatic and non-traumatic loads (Shoham and Gefen, 2012; Valdez and Balachandran, 2013) End-organ damage due to blood pressure (Safar et al., 2012) Inflammation (Abolhassani et al., 2008; Schwartz et al., 2008; Levy Nogueira et al., 2016) Cancer (Schwartz et al., 2002; Schwartz, 2004; Levy Nogueira et al., 2016)	Avula, 1994; Schwartz et al., 2002; Schwartz, 2004; Abolhassani et al., 2008; Safar et al., 2012; Shoham and Gefen, 2012; Valdez and Balachandran, 2013; Bloomfield et al., 2016; Johnson and Luks, 2016; Levy Nogueira et al., 2016	Schwartz et al., 2002, 2008; Schwartz, 2004; Safar et al., 2012; Levy Nogueira et al., 2016

* *Fluid shifts: edema, ascites, pleural, pericardial and joint effusion*.

** *Numbers in square are the related references*.

Fluid mechanical constraints can also be caused by hemodynamic stress. Hydrodynamic forces acting on vessel walls include shear stress generated by blood flow and circumferential stress resulting from blood pressure. Morphological and molecular changes in blood vessels ascribed to elevated pressure consist of endothelial damage, neointima formation, activation of inflammatory cascades, uptake of atherogenic lipoproteins, hypertrophy, migration and changes in vascular smooth muscle cells, as well as extracellular matrix imbalances (Cunningham and Gotlieb, 2005; Anwar et al., 2012). The exact mechanism of converting shear energy into biochemical signal is not yet well understood. The strain in atherosclerotic plaques due to the pulsatile pressure is highly correlated to plaque rupture and thus an ischemic event (De Korte et al., 2016). Hemodynamic stress also induces cardiac valve disease (Robicsek and Thubrikar, 2002), ventricular hypertrophy (Neeland et al., 2013), pulmonary hypertension (Puwanant et al., 2010), brain lacunar infarcts, microbleeds, and white matter hyperintensities (Saji et al., 2016), as well as glomerulopathy (Gnudi et al., 2003).

Chronic adult hydrocephalus is characterized by an excessive enlargement of the brain ventricles, which leads to parenchymal shrinkage. It is most commonly accepted that continuous or transient cerebrospinal fluid hypertension leads to chronic hydrodynamic stress on ventricular walls, ultimately resulting in ventricular dilatation (Streitberger et al., 2011). Hemodynamic stress has also associated with migraine (van Alphen, 1986; Gudmundsson et al., 2006). Epidemiological, neuropathological, microstructural studies largely support the notion that mechanical stress triggers and/or accelerates neurodegenerative diseases, including AD, Parkinson' disease and amyotrophic lateral sclerosis. Hemodynamic and hydrodynamic factors, such as hypertension and chronic adult hydrocephalus, and exposition to traumatic brain injury consists on well-established risk factors for AD (Plassman et al., 2000; Schmidt et al., 2001; Goldman et al., 2006; Uryu et al., 2007; Johnson et al., 2012; Levy Nogueira et al., 2016). Finally, energy storage and dissipation, explained by the laws of solid mechanics, are related to possible mechanisms of changes in cartilage structure and function that occur in osteoarthritis (Silver and Bradica, 2002; Vincent et al., 2012; Visser et al., 2014).

### Diseases exhibiting changes in electrical features: electric phenotypes

Depression and neurodegenerative disorders are characterized by reduced electrical brain excitability (Concerto et al., 2013; Ni and Chen, 2015) (Table 2). For this reason, numerous studies have addressed short-term and long-term effects of transcranial direct current stimulation (tDCS) (Meinzer et al., 2014), transcranial magnetic stimulation (TMS) (Jay et al., 2016), electroconvulsive therapy (ECT), and deep brain stimulation (DBS) on these affections (Kumar et al., 1998). Bradyarrhythmias could also be classified as diseases with reduced electrical currents (Song et al., 2012). They have been classically treated by cardiac pacemaker stimulation. Epilepsy (Reynolds, 2001), restless legs syndrome (Bara-Jimenez et al., 2000), pain disorders (Theuvenet et al., 1999), bipolar disorder (Mertens et al., 2015), essential tremor (Louis, 2014), cardiac tachyarrhythmias (Li et al., 2009) (e.g., atrial fibrillation, flutter) are diseases characterized by electrical hyperexcitability. They have been treated by drugs that modulate ion channels, such as antiepileptic drugs, benzodiazepines and antiarrhythmic agents (Katzung et al., 2015). They target transmembrane channels modulating transmembrane currents.

**Table 2:** Electric phenotypes of diseases.

**Electrical phenotype**	**Decreased**	**Increased**	**Electrical forces, energy, potentials, currents, discharges, excitability**
Skeletal muscle and nerve	Myopathies Neuropathies Channelopathies Myasthenia gravis	Muscle spasticity, tetany and reinnervation Neuropathic pain	Gutmann and Gutmann, 1996; Theuvenet et al., 1999; Preston and Shapiro, 2012; Sheean, 2012; Abraham et al., 2016
Cardiac muscle	Bradyarrhythmias	Tachyarrhythmias (flutter, atrial fibrillation)	Li et al., 2009; Song et al., 2012
Brain and spinal cord	Depression Neurodegenerative diseases Coma and brain death	Bipolar disorder Restless legs syndrome Epilepsy Essential tremor	Bara-Jimenez et al., 2000; Concerto et al., 2013; Mertens et al., 2015; Ni and Chen, 2015 Reynolds, 2001; Louis, 2014
Cancer	Mitochondrial dysfunction (Warburg effect)		Pokorný et al., 2014
Therapies	Drugs: antiepileptics, neuroleptics benzodiazepines, anesthetics, muscle relaxants, antiarrhythmics	Transcranial direct current stimulation (tDCS) Transcranial magnetic stimulation (TMS) Deep brain stimulation (DBS) Electroconvulsive therapy (ECT)	Kumar et al., 1998; Meinzer et al., 2014; Katzung et al., 2015; Jay et al., 2016

### Thermodynamic phenotypes of cancer

In the case of cancer there is concomitant increased pressure and decreased electromagnetic fields. During hepatic biopsy, the interstitial pressure of the hepatic parenchyma was measured. About 17–19 gauge guiding needle was advanced to the tumor under CT guidance (Schwartz, 2014). The pressure of the normal liver parenchyma is 4 mm Hg. It raises to 13 in premalignant cirrhosis. The pressure of primary liver cancer was between 25 and 26 mm Hg. The increased pressure may be a direct consequence of impaired mitochondrial activity such as described by Otto Warburg (Schwartz et al., 2017) (Table 3).

**Table 3:** Thermodynamic phenotypes of the diseases.

**Thermodynamic phenotype**	**Decreased**	**Increased**	**Metabolic rate**
Lifespan	Aging	Childhood	Demetrius et al., 2014
Consciousness level	Sleep, delirium, torpor, coma	Wakefulness	Staples, 2016
Physical activity	Rest	Exercise	
Systemic conditions	Hypothyroidism Metabolic syndrome Malnutrition Anemia Ischemia Drugs: beta blockers	Hyperthyroidism Fever Burn injury Drugs: thyroid hormones, metformin, epinephrine and other adrenergic agonists	Magnus-Levy, 1895; Nahorski and Rogers, 1973; Fisher et al., 1998; Ratheiser et al., 1998; Singhal et al., 2002; Emery, 2005; Porter et al., 2014
Cancer	Mitochondrial OxPhos^*^ -Therapeutic approach: ↗ mitochondrial OxPhos^*^	-Cytosolic glucose metabolism^*^ -Therapeutic approach: ↘ cytosolic glucose metabolism	Warburg, 1956; Levine and Puzio-Kuter, 2010; Schwartz et al., 2010; Israël and Schwartz, 2011; Porporato et al., 2011; Abolhassani et al., 2012
Neuropsychiatric conditions	Alzheimer's disease: -Cytosolic glucose metabolism^**^ Parkinson's disease Huntington disease Schizophrenia	Alzheimer's disease: -Mitochondrial OxPhos^**^ Epilepsy Drugs: amphetamines	Hirai et al., 2001; Beal, 2005; Moreira et al., 2007; Narendra et al., 2009; Clay et al., 2011 Yakushev et al., 2011; Demetrius and Driver, 2013; Demetrius et al., 2014

* *Warburg effect*,

** *Inverse Warburg effect, ↗ stimulate, ↙inhibit. OxPhos, oxidative phosphorylation*.

## Mechanical, electric, and chemical phenotypes are intertwined

The different forms of energy are interconvertible, following James Joule's discovery (Joule, 1843). The first law of thermodynamics is a statement regarding the conservation of energy: although energy can be converted from one form to another, the total energy of a closed isolated system is constant. Inorganic as well as living organisms are following the rules of thermodynamics. They are most of the time in a non-equilibrium state, but are very often in a stationary state. But in general for local analysis (in the order of cell size) or for stationary state one may describe their behavior as a near-equilibrium system, using therefore equilibrium thermodynamics variables. Diseases could be explained by their departure from a homeostatic stationary state (Demetrius, 2013). Consequently, mechanical, electric and chemical energies are intertwined in metabolism of living organisms. As stated before, cancer is related to increased interstitial and intracellular pressure and decreased chemical rate of ATP formation within mitochondria (Warburg effect). Inflammation is also linked to increased osmotic pressure, a way to translate chemical gradients into pseudo-mechanical driving forces, and a transient Warburg effect, a partial and reversible inhibition of mitochondrial activity (Srivastava and Mannam, 2015; Aounallah et al., 2016). In both cancer and inflammation situations, energy is redirected from oxidative phosphorylation (mitochondria) to the pentose phosphate pathway (cytosol), increasing biomass synthesis and, theoretically, intracellular osmotic pressure and colloid pressure (aka oncotic pressure, a special osmotic pressure due to the presence of colloidal proteins in the blood plasma).

**Figure 1:**
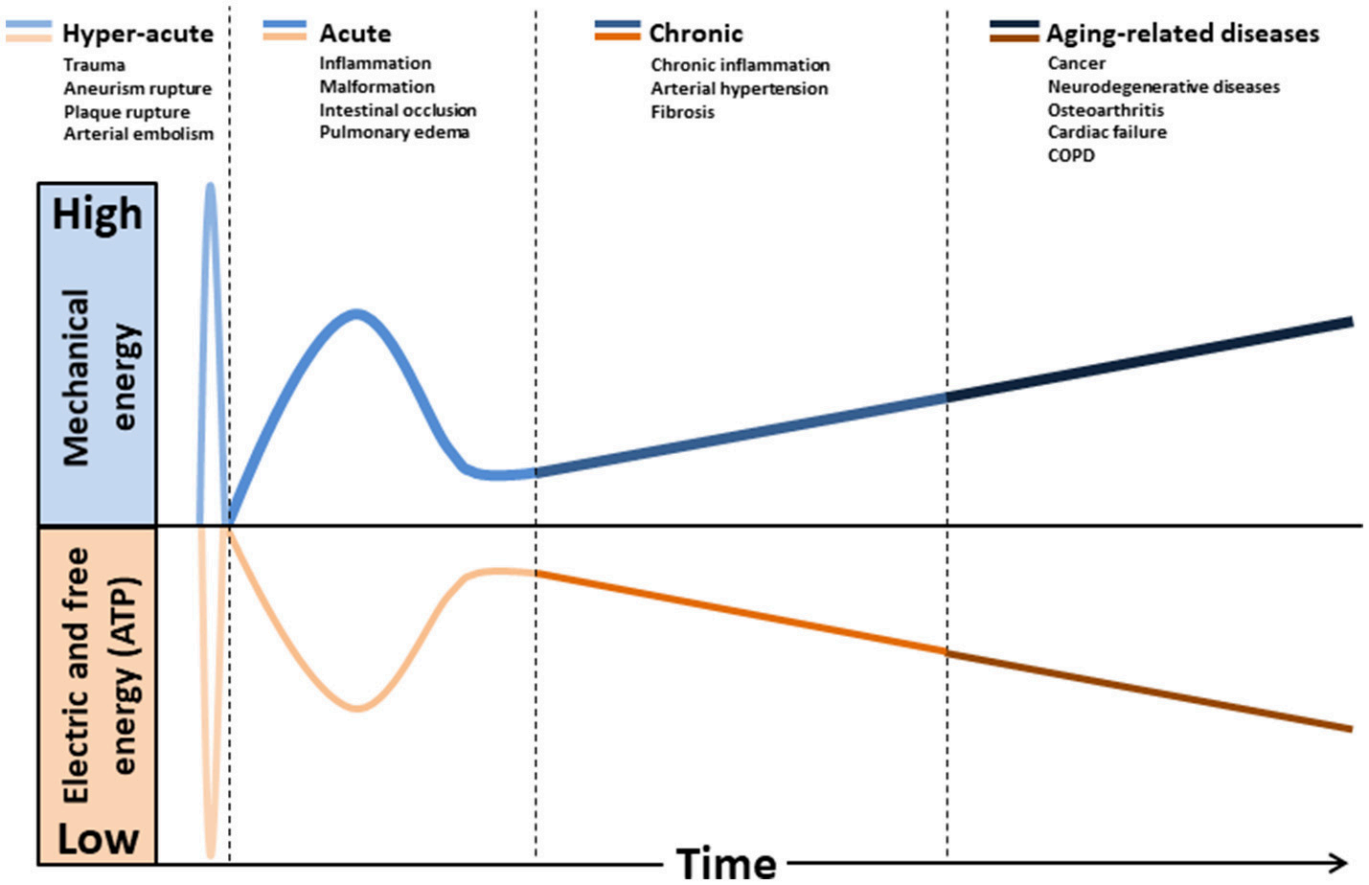
Hypothetical model relating the natural history of diseases to mechanical stress accumulation coupled to electric forces and free energy (ATP) decline. Intra and extracellular long-term consequences of mechanical stress imply in deformation and/or breakdown of extracellular matrix, cytoskeleton, and membranes (including mitochondria). Consequently, transmembrane plasmatic and mitochondrial electric potentials decline. The metabolic rate of generation of free energy through ATP also declines, due to its dependence to mitochondrial inner membrane integrity. Glycolysis will be abnormally up-regulated (Warburg effect), as it does not depends on mitochondrial membranes. This mode of fermentation generates thermodynamically stable biomass, composed by, for example, phospholipid membranes, nucleic acid (proliferation) and cellular waste aggregates (fibrosis, brain protein deposits).

The effect of mechanical forces on mitochondrion has been poorly studied. This organelle is composed essentially of soft bilayer membranes and many of its functions involve the manipulation of its curvature, as it is easy to sustain curvature strains in a membrane due to its high elasticity (Kumar et al., 1998). Differences in tension between the two membrane interfaces can create changes in curvature with the displacement of lipids, channels, and pumps. As a consequence, the resultant of the electrical forces across curved membranes can change (Petrov, 2006). This phenomenon is called flexoelectric effect (Petrov, 2002, 2006) and it explains, for example, how mechanosensing organelles of hair cells respond to the fluid motion in the inner ear, converting membrane deformation into electric signals. Conformational changes induced by cytoskeletal tension or osmotic pressure may convert mitochondrion into a non-energized state, impairing electrical currents, but still allowing mitochondrial smooth movements of fission and fusion. The fact that energized mitochondria have inner membranes extensively curved is quite indicative of the role of flexoelectricity in the energy transformation (Hackenbrock, 1968; Harris et al., 1968; Green and Young, 1971). Indeed, thermodynamic laws have predicted that membrane tension modulates transmembrane voltage (Zhang et al., 2001). Figure 1 shows a hypothetical model relating the natural history of diseases to mechanical stress accumulation, electric forces, and chemical energy (ATP).

## Handling the complexity of phenotypes in a single frame

Up to now, single components of phenotypes have been reported, as well as intertwined components. For each category: mechanical, electric, and chemical physico-chemical phenotypes a wide variety of incarnations exists. Each component of a phenotype is characterized by its free energy, and the global system described is a full thermodynamic system. Physico-chemical phenotypes can be extended to any sort of energy containing interaction as is immediately understandable for magnetic or light (electromagnetic) or radiations interactions. MRI, Radiotherapy, laser medicine are common applications of using such means of interactions with physical constraint. Each energy component of a phenotype can involve several intensive parameters. An energy may arise from simultaneous action of two metabolic pathways coupling two chemical reactions. Another may involve **p**ressure and a chemical gradient, etc. For each doubly, triply or most of the time more coupled interactions, a much wider variety of components exist. Would it be therefore possible to combine them in order to profile diseases from the phenotypic point of view? This may be a way to act onto proper variables in order to reprogram them and to restore the homeostatic state.

Biological systems when modeled as physical systems are thermodynamic open systems exhibiting a hierarchical organization. Therefore the global system must be represented by sub-systems that are weakly connected and can each be considered as an open system itself. When in a stationary state, they may be treated as at equilibrium, and the variables to manipulate each sub-system can be those of equilibrium. Therefore, such a frame offers the ability to organize phenotypes by their disease profile, a successful strategy used by Damasio in his research about the structure of the brain called the “human lesion method.” In such a strategy the number of variables modified by the existence of the lesion is much lower than the total number of variables necessary to describe the overall thermodynamic system.

Therefore if a system of objects is to be analyzed, each component or entity like a cell or any relevant organ or organelle is to be thermodynamically described. Its free energy can be described as the sum of the free energy of a standard state plus the sum of all additional free energy components needed to describe their departure from the standard state. Chemical electric and mechanical energies, as well as magnetic and electromagnetic (light) energies, must be considered for each node of the graph of the subsystem. Moving from point approximation for electrons or atoms to molecules or ions, the space extension of larger objects must be take into account. This is done by attaching to each node a shape. Therefore energies that are expressed in their simple point version as product of numbers (ex: charge, voltage) become spatial extensions described by geometrical “tensors.” Point objects are rank 0 tensors, a rod or wire a rank 1 tensor, an ellipsoidal object (spherical cell or oblong cell) a rank 2 tensor and so on. Most of simple products like an electric energy zi.Φi become tensor products {zi}⊗{Φ_i_}. Here a charge tensor and an electric field tensor, each of rank 2. Standard states can be chosen as the stationary states, or homeostatic standard states.

Once this set of axiomatic choice has been made then a disease will be described by all the functional components that are strongly linked leading to a subset of the large system, leaving the weak linkages outside the primary scope of description. The variables attached to the nodes of the graph as well as the graph are the representation of the physico-chemical phenotype of the disease.

In the current state of such “network medicine” formalism the current phenotypes based on non-physico-chemical elements are inadequate (Loscalzo et al., 2007). However, if molecular entities are taken into account it is surprising that physical parameters seem paradoxically to be completely absent from the postgenomic era approach. Electric, magnetic field, T, P have to be added in a thermodynamic system. Furthermore, these physical parameters might not be constant, but variable, as for example a radio-frequency electric or magnetic field. Cellular phones, all sorts of wireless connections, are generating such frequencies and are definitely part of a phenotype. Pressure pulsations are generated by acoustic waves etc. All free energies available for describing the thermodynamic model in phenotypes, not only for assessing the disease but addressing the therapeutical strategies, may contain all sort of physical energy contributions creating much more connections between nodes than the simple molecular genetic or metabolic pathways.

To face such a complexity, hierarchization of the system is mandatory. However this hierarchization must fulfill one essential criterion: phenotypes must be as mentioned earlier at the same time able to group, within a limited number of categories, a large number of diseases and must be usable by practitioners to define a possible strategy for curing the disease. Most of graph theories developed up to now are focused on molecular and genetic approaches giving rise to metabolomics, genomics and other typologies but they are missing several of the critical criteria that could them make usable by the researcher as well as the practitioner. Among the obvious missing pieces of information, physical phenotypic parameters are not considered may be useful for medical doctors in his/her therapeutic guidance.

The purpose of this contribution is to propose another vision of describing diseases by using a set of phenotypes analogous to Mendeleev's table (Table 1). This would give rise to an infinite possibility of combinations with a reasonable small set of phenotypes and to a very large set of rules leading to an infinite number of diseases, analogous to the extremely large scope of chemistry in all its incarnations.

## Conclusion and perspectives

To advance our understanding of the mechanisms of diseases, biological process needs to be evaluated and integrated into more comprehensive and global theories, accounting with principles of physics. The word “physician” is a reminiscence of the time when medicine was a part of Physics. We propose a conceptual framework that outlines integrative approaches to classify diseases based on physics-based phenotypes. Classical diagnostic systems may not capture mechanical, electric and thermodynamic underlying common mechanisms of diseases. Identifying syndromes based on these phenotypes may improve therapeutic outcomes. For example, electrical energy is a treatment for numerous brain disorders via (tDCS) (Meinzer et al., 2014), TMS (Jay et al., 2016), electroconvulsive therapy (ECT), and DBS (Kumar et al., 1998). The present perspective toward an innovative disease classification may also improve drug-repurposing strategies in the future. We recognize that we are still a long way from knowing if this approach will succeed. The physical parameters should be introduced in “omics” studies and especially correlations between parameters governing basic thermodynamic behavior. Such correlations affect many processes at a time, and must be extremely useful to correlate with other metabolic profiling methods.

## Author contributions

LS and OL: elaborated the concept of this article and wrote the article; JdVM: reviewed and wrote the article.

### Conflict of interest statement

The authors declare that the research was conducted in the absence of any commercial or financial relationships that could be construed as a potential conflict of interest.

## References

[B1] Abolhassani M. Guais A. Sanders E. Campion F. Fichtner I. Bonte J.. (2012). Screening of well-established drugs targeting cancer metabolism: reproducibility of the efficacy of a highly effective drug combination in mice. Invest. New Drugs 30, 1331–1342. 10.1007/s10637-011-9692-7 21655919

[B2] Abolhassani M. Wertz X. Pooya M. Chaumet-Riffaud P. Guais A. Schwartz L. (2008). Hyperosmolarity causes inflammation through the methylation of protein phosphatase 2A. Inflamm. Res. 57 419–429. 10.1007/s00011-007-7213-0 18777115

[B3] Abraham A. Alabdali M. Alsulaiman A. Breiner A. Barnett C. Katzberg H. D. (2016). Repetitive nerve stimulation cut-off values for the diagnosis of myasthenia gravis. Muscle Nerve. 55, 166–170. 10.1002/mus.25214 27287989

[B4] Abramson S. B. Attur M. (2009). Developments in the scientific understanding of osteoarthritis. Arthritis Res. Ther. 11, 227. 10.1186/ar2655 19519925 PMC2714096

[B5] Anwar M. A. Shalhoub J. Lim C. S. Gohel M. S. Davies A. H. (2012). The effect of pressure-induced mechanical stretch on vascular wall differential gene expression. J. Vasc. Res. 49, 463–478. 10.1159/000339151 22796658

[B6] Aounallah M. Dagenais-Lussier X. El-Far M. Mehraj V. Jenabian M. A. van Grevenynghe J.. (2016). Current topics in HIV pathogenesis, part 2: inflammation drives a Warburg-like effect on the metabolism of HIV-infected subjects. Cytokine Growth Factor Rev. 28, 1–10. 10.1016/j.cytogfr.2016.01.001 26851985

[B7] Avula X. J. (1994). Simulation of gravitational field variation on fluid-filled biological membranes. J. Gravitational Physiol. J. Int. Soc. Gravit. Physiol. 1, P108–P109. 11538733

[B8] Bara-Jimenez W. Aksu M. Graham B. Sato S. Hallett M. (2000). Periodic limb movements in sleep: state-dependent excitability of the spinal flexor reflex. Neurology 54, 1609–1616. 10.1212/WNL.54.8.1609 10762502

[B9] Beal M. F. (2005). Mitochondria take center stage in aging and neurodegeneration. Ann. Neurol. 58, 495–505. 10.1002/ana.20624 16178023

[B10] Bernard C. (1865). Introduction à L'étude de la Médecine Expérimentale. Paris: J. B. Baillière et fils.

[B11] Bernard C. (2013). Lecons de Physiologie Experimentale Appliquee a la Medecine, Faites Au College de France. Paris: Hachette Livre - Bnf.

[B12] Bidan C. M. Veldsink A. C. Meurs H. Gosens R. (2015). Airway and extracellular matrix mechanics in COPD. Front. Physiol. 6:346. 10.3389/fphys.2015.00346 26696894 PMC4667091

[B13] Blake G. J. Ridker P. M. (2002). Inflammatory bio-markers and cardiovascular risk prediction. J. Intern. Med. 252 283–294. 10.1046/j.1365-2796.2002.01019.x 12366601

[B14] Bloomfield S. A. Martinez D. A. Boudreaux R. D. Mantri A. V. (2016). Microgravity stress: bone and connective tissue. Compr. Physiol. 6, 645–686. 10.1002/cphy.c130027 27065165

[B15] Boron W. F. (2004). Regulation of intracellular pH. Adv. Physiol. Educ. 28, 160–179. 10.1152/advan.00045.2004 15545345

[B16] Brinckmann P. Frobin W. Leivseth G. (2002). Musculoskeletal Biomechanics. Munster: Thieme.

[B17] Busa W. B. Crowe J. H. (1983). Intracellular pH regulates transitions between dormancy and development of brine shrimp (*Artemia salina*) embryos. Science 221, 366–368. 10.1126/science.221.4608.366 17798891

[B18] Busa W. B. Crowe J. H. Matson G. B. (1982). Intracellular pH and the metabolic status of dormant and developing Artemia embryos. Arch. Biochem. Biophys. 216, 711–718. 10.1016/0003-9861(82)90261-2 7114857

[B19] Butera R. J. Rinzel J. Smith J. C. (1999). Models of respiratory rhythm generation in the pre-Bötzinger complex. I. Bursting pacemaker neurons. J. Neurophysiol. 82, 382–397. 10.1152/jn.1999.82.1.382 10400966

[B20] Carter K. C. (1980). Germ theory, hysteria, and Freud's early work in psychopathology. Med. Hist. 24, 259–274. 10.1017/S002572730004031X 6997653 PMC1082654

[B21] Celli B. R. (2002). Respiratory management of diaphragm paralysis. Semin. Respir. Crit. Care Med. 23, 275–281. 10.1055/s-2002-33036 16088620

[B22] Charlton M. R. Nair K. S. (1998). Role of hyperglucagonemia in catabolism associated with type 1 diabetes: effects on leucine metabolism and the resting metabolic rate. Diabetes 47, 1748–1756. 10.2337/diabetes.47.11.1748 9792544

[B23] Christen R. Schackmann R. W. Shapiro B. M. (1983). Metabolism of sea urchin sperm. Interrelationships between intracellular pH, ATPase activity, and mitochondrial respiration. J. Biol. Chem. 258, 5392–5399. 6222053

[B24] Clay H. B. Sillivan S. Konradi C. (2011). Mitochondrial dysfunction and pathology in bipolar disorder and schizophrenia. Int. J. Dev. Neurosci. 29, 311–324. 10.1016/j.ijdevneu.2010.08.007 20833242 PMC3010320

[B25] Concerto C. Lanza G. Cantone M. Pennisi M. Giordano D. Spampinato C.. (2013). Different patterns of cortical excitability in major depression and vascular depression: a transcranial magnetic stimulation study. BMC Psychiatry 13:300. 10.1186/1471-244X-13-300 24206945 PMC4226249

[B26] Cunningham K. S. Gotlieb A. I. (2005). The role of shear stress in the pathogenesis of atherosclerosis. Lab. Invest. 85, 9–23. 10.1038/labinvest.3700215 15568038

[B27] da Veiga Moreira J. Peres S. Steyaert M-J. Bigan E. Paulevé L. Schwartz L.. (2015). Cell cycle progression is regulated by intertwined redox oscillators. Theor. Biol. Med. Model. 12:10. 10.1186/s12976-015-0005-2 26022743 PMC4459109

[B28] De Korte C. L. Fekkes S. Nederveen A. J. Manniesing R. Hansen H. G. (2016). Review: Mechanical Characterization of Carotid Arteries and Atherosclerotic Plaques. IEEE Trans. Ultrason. Ferroelectr. Freq. Control. 10.1109/TUFFC.2016.2572260 27249826

[B29] DeDuve C. (1991). Blueprint for a Cell: the Nature and Origin of Life, 1 edn, Burlington, NC: Carolina Biological Supply Co.

[B30] Demetrius L. A. (2013). Boltzmann, darwin and directionality theory. Phys. Rep. 530, 1–85. 10.1016/j.physrep.2013.04.001

[B31] Demetrius L. A. Driver J. (2013). Alzheimer's as a metabolic disease. Biogerontology 14, 641–649. 10.1007/s10522-013-9479-7 24249045

[B32] Demetrius L. A. Coy J. F. Tuszynski J. A. (2010). Cancer proliferation and therapy: the Warburg effect and quantum metabolism. Theor. Biol. Med. Model. 7:2. 10.1186/1742-4682-7-2 20085650 PMC2819045

[B33] Demetrius L. A. Magistretti P. J. Pellerin L. (2014). Alzheimer's disease: the amyloid hypothesis and the Inverse Warburg effect. Front. Physiol. 5:522. 10.3389/fphys.2014.00522 25642192 PMC4294122

[B34] Diamant N. E. (2006). Pathophysiology of Gastroesophageal Reflux Disease. GI Motil Online. 10.1038/gimo21

[B35] Douville N. J. Zamankhan P. Tung Y. C. Li R. Vaughan B. L. Takayama C. S.. (2011). Combination of fluid and solid mechanical stresses contribute to cell death and detachment in a microfluidic alveolar model. Lab. Chip 11, 609–619. 10.1039/C0LC00251H 21152526

[B36] Eiken O. Kölegård R. Mekjavic I. B. (2008). Pressure-distension relationship in arteries and arterioles in response to 5 wk of horizontal bedrest. Am. J. Physiol. Heart Circ. Physiol. 295, H1296–H1302. 10.1152/ajpheart.00576.2008 18660441

[B37] Emery P. W. (2005). Metabolic changes in malnutrition. Eye Lond. Engl. 19, 1029–1034. 10.1038/sj.eye.6701959 16304580

[B38] Eyckmans J. Boudou T. Yu X. Chen C. S. (2011). A hitchhiker's guide to mechanobiology. Dev. Cell. 21, 35–47. 10.1016/j.devcel.2011.06.015 21763607 PMC3155761

[B39] Fang B. Wang D. Huang M. Yu G. Li H. (2010). Hypothesis on the relationship between the change in intracellular pH and incidence of sporadic Alzheimer's disease or vascular dementia. Int. J. Neurosci. 120, 591–595. 10.3109/00207454.2010.505353 20707633

[B40] Fermi E. (1956). Thermodynamics. New York, NY: Dover Publications.

[B41] Fisher M. H. Amend A. M. Bach T. J. Barker J. M. Brady E. J. Candelore M. R.. (1998). A selective human β3 adrenergic receptor agonist increases metabolic rate in rhesus monkeys. J. Clin. Invest. 101, 2387–2393. 10.1172/JCI2496 9616210 PMC508828

[B42] Flammer J. Orgül S. Costa V. P. Orzalesi N. Krieglstein G. K. Serra L. M.. (2002). The impact of ocular blood flow in glaucoma. Prog. Retin. Eye Res. 21, 359–393. 10.1016/S1350-9462(02)00008-3 12150988

[B43] Fleury V. Schwartz L. (2003). Numerical investigation of the effect of loss of cellular polarity on cancer invasiveness and geometry. Fractals 11, 397–414. 10.1142/S0218348X0300204X

[B44] Frankenfield D. C. Smith J. S. Cooney R. N. Blosser S. A. Sarson G. Y. (1997). Relative association of fever and injury with hypermetabolism in critically ill patients. Injury 28, 617–621. 10.1016/S0020-1383(97)00117-4 9624339

[B45] Gnudi L. Viberti G. Raij L. Rodriguez V. Burt D. Cortes P.. (2003). GLUT-1 overexpression: link between hemodynamic and metabolic factors in glomerular injury? Hypertension 42, 19–24. 10.1161/01.HYP.0000075949.19968.EF 12771048

[B46] Goldman D. E. (1943). Potential, impedance and rectification in membranes. J. Gen. Physiol. 27, 37–60. 10.1085/jgp.27.1.37 19873371 PMC2142582

[B47] Goldman S. M. Tanner C. M. Oakes D. Bhudhikanok G. S. Gupta A. Langston J. W. (2006). Head injury and Parkinson's disease risk in twins. Ann. Neurol. 60, 65–72. 10.1002/ana.20882 16718702

[B48] Gottlieb R. A. Nordberg J. Skowronski E. Babior B. M. (1996). Apoptosis induced in Jurkat cells by several agents is preceded by intracellular acidification. Proc. Natl. Acad. Sci. U.S.A. 93, 654–658. 10.1073/pnas.93.2.654 8570610 PMC40107

[B49] Green D. E. Young J. H. (1971). Energy transduction in membrane systems. Am. Sci. 59, 92–100. 5547538

[B50] Gregory D. E. Callaghan J. P. (2010). An examination of the influence of strain rate on subfailure mechanical properties of the annulus fibrosus. J. Biomech. Eng. 132:091010. 10.1115/1.4001945 20815644

[B51] Grimble R. F. (2003). Inflammatory response in the elderly. Curr. Opin. Clin. Nutr. Metab. Care 6, 21–29. 10.1097/00075197-200301000-00005 12496677

[B52] Gudmundsson L. S. Thorgeirsson G. Sigfusson N. Sigvaldason H. Johannsson M. (2006). Migraine patients have lower systolic but higher diastolic blood pressure compared with controls in a population-based study of 21,537 subjects. The Reykjavik Study. Cephalalgia Int. J. Headache 26, 436–444. 10.1111/j.1468-2982.2005.01057.x 16556245

[B53] Gutmann L. Gutmann L. (1996). Axonal channelopathies: an evolving concept in the pathogenesis of peripheral nerve disorders. Neurology 47, 18–21. 10.1212/WNL.47.1.18 8710074

[B54] Joule J. P. (1843). On the Calorific Effects of Magneto-Electricity, and on the Mechanical Value of Heat. Avaliable online at: http://iom3.tandfonline.com/doi/abs/10.1080/14786444308644730?journalCode=tphm14

[B55] Hackenbrock C. R. (1968). Ultrastructural bases for metabolically linked mechanical activity in mitochondria ii. electron transport-linked ultrastructural transformations in mitochondria. J. Cell Biol. 37, 345–369. 10.1083/jcb.37.2.345 5656397 PMC2107416

[B56] Hamill O. P. Martinac B. (2001). Molecular basis of mechanotransduction in living cells. Physiol. Rev. 81, 685–740. 10.1152/physrev.2001.81.2.685 11274342

[B57] Hand S. C. Carpenter J. F. (1986). pH-induced metabolic transitions in artemia embryos mediated by a novel hysteretic trehalase. Science 232, 1535–1537. 10.1126/science.232.4757.1535 17773504

[B58] Harris R. A. Penniston J. T. Asai J. Green D. E. (1968). The conformational basis of energy conservation in membrane systems. II. Correlation between conformational change and functional states. Proc. Natl. Acad. Sci. U.S.A. 59, 830–837. 10.1073/pnas.59.3.830 5238663 PMC224758

[B59] Hasiloglu Z. I. Albayram S. Gorucu Y. Selcuk H. Cagil E. Erdemli H. E.. (2012). Assessment of CSF flow dynamics using PC-MRI in spontaneous intracranial hypotension. Headache 52, 808–819. 10.1111/j.1526-4610.2012.02150.x 22512384

[B60] Henderson W. R. Sheel A. W. (2012). Pulmonary mechanics during mechanical ventilation. Respir. Physiol. Neurobiol. 180, 162–172. 10.1016/j.resp.2011.11.014 22154694

[B61] Hirai K. Aliev G. Nunomura A. Fujioka H. Russell R. L. Atwood C. S.. (2001). Mitochondrial abnormalities in Alzheimer's disease. J. Neurosci. 21, 3017–3023. 11312286 10.1523/JNEUROSCI.21-09-03017.2001 PMC6762571

[B62] Hodgkin A. L. Huxley A. F. (1952). A quantitative description of membrane current and its application to conduction and excitation in nerve. J. Physiol. 117, 500–544. 10.1113/jphysiol.1952.sp004764 12991237 PMC1392413

[B63] Holcman D. Yuste R. (2015). The new nanophysiology: regulation of ionic flow in neuronal subcompartments. Nat. Rev. Neurosci. 16, 685–692. 10.1038/nrn4022 26462753

[B64] Imray C. (2016). Lessons from altitude: cerebral perfusion insights and their potential clinical significance. Exp. Physiol. 101, 1167–1172. 10.1113/EP085813 27061345

[B65] Israël M. Schwartz L. (2011). The metabolic advantage of tumor cells. Mol. Cancer 10:70. 10.1186/1476-4598-10-70 21649891 PMC3118193

[B66] Jacobs C. R. Huang H. Kwon R. Y. (2012). Introduction to Cell Mechanics and Mechanobiology. New York, NY: Garland Science.

[B67] Jay E.-L. Nestler S. Sierra M. McClelland J. Kekic M. David A. S. (2016). Ventrolateral prefrontal cortex repetitive transcranial magnetic stimulation in the treatment of depersonalization disorder: a consecutive case series. Psychiatry Res. 240, 118–122. 10.1016/j.psychres.2016.04.027 27104926 PMC4906152

[B68] Jiang G. Solbrig H. R. Chute C. G. (2013). Using semantic Web technology to support icd-11 textual definitions authoring. J. Biomed. Semant. 4:11. 10.1186/2041-1480-4-11 23601451 PMC3653695

[B69] Johnson N. J. Luks A. M. (2016). High-altitude medicine. Med. Clin. North Am. 100, 357–369. 10.1016/j.mcna.2015.09.002 26900119

[B70] Johnson V. E. Stewart W. Smith D. H. (2012). Widespread τ and amyloid-β pathology many years after a single traumatic brain injury in humans. Brain Pathol. Zurich Switz. 22, 142–149. 10.1111/j.1750-3639.2011.00513.x 21714827 PMC3979351

[B71] Katzung B. G. Masters S. B. Trevor A. J. (2015). Basic & Clinical Pharmacology, 13th edn. San Francisco, CA: McGraw-Hill Professional.

[B72] Kim H.-D. Wong P. K. Park J. Levchenko A. Sun Y. (2009). Microengineered platforms for cell mechanobiology. Annu. Rev. Biomed. Eng. 11, 203–233. 10.1146/annurev-bioeng-061008-124915 19400708

[B73] Kongstad L. Grände P. O. (1999). Local vascular response during organ elevation. A model for cerebral effects of upright position and dural puncture. Acta Anaesthesiol. Scand. 43. 438–446. 10.1034/j.1399-6576.1999.430412.x 10225078

[B74] Kreimeier U. (2000). Pathophysiology of fluid imbalance. Crit. Care 4, S3–S7. 10.1186/cc968 11255592 PMC3226173

[B75] Kubota K. Yoshimura N. Yokota M. Fitzsimmons R. J. Wikesjö M. E. (1995). Overview of effects of electrical stimulation on osteogenesis and alveolar bone. J. Periodontol. 66, 2–6. 10.1902/jop.1995.66.1.2 7891245

[B76] Kumar R. Lozano A. M. Kim Y. J. Hutchison W. D. Sime E. Halket E.. (1998). Double-blind evaluation of subthalamic nucleus deep brain stimulation in advanced Parkinson's disease. Neurology 51, 850–855. 10.1212/WNL.51.3.850 9748038

[B77] Kurdistani S. K. (2014). Chromatin: a capacitor of acetate for integrated regulation of gene expression and cell physiology. Curr. Opin. Genet. Dev. 26, 53–58. 10.1016/j.gde.2014.06.002 25016437 PMC4253552

[B78] Lane N. Martin W. F. (2012). The origin of membrane bioenergetics. Cell 151, 1406–1416. 10.1016/j.cell.2012.11.050 23260134

[B79] Lee C. H. Cragoe E. J. Edwards A. M. (2003). Control of hepatocyte DNA synthesis by intracellular pH and its role in the action of tumor promoters. J. Cell. Physiol. 195, 61–69. 10.1002/jcp.10225 12599209

[B80] Lehninger A. L. (1965). Bioenergetics: The Molecular Basis of Biological Energy Transformations. New York, NY: Benjamin/Cummings Pub.

[B81] Lehninger A. L. (1971). Bioenergetics in Soft Cover - “Pursuit of Happiness” Books, W.A. Benjamin Publishing Avaliable online at: http://www.abebooks.com/Bioenergetics-Albert-L-Lehninger-W.A-Benjamin/401866590/bd (Accessed May 1, 2016).

[B82] Levine A. J. Puzio-Kuter A. M. (2010). The control of the metabolic switch in cancers by oncogenes and tumor suppressor genes. Science 330, 1340–1344. 10.1126/science.1193494 21127244

[B83] Levy Nogueira M. da Veiga Moreira J. Baronzio G. F. Dubois B-J. Steyaert M. Schwartz L. (2016). Mechanical Stress as the Common denominator between chronic inflammation, cancer, and Alzheimer's Disease. Front. Oncol. 5:197. 10.3389/fonc.2015.00197 26442209 PMC4585184

[B84] Levy Nogueira M. Epelbaum S. Steyaert J-M. Dubois B. Schwartz L. (2015a). Mechanical stress models of Alzheimer's disease pathology. Alzheimers Dement. J. Alzheimers Assoc. 12, 324–333. 10.1016/j.jalz.2015.10.005 26718585

[B85] Levy Nogueira M. Lafitte O. Steyaert J-M. Bakardjian H. Dubois B. Schwartz L.. (2015b). Mechanical stress related to brain atrophy in *Alzheimer's* disease. Alzheimers Dement. 12, 11–20. 10.1016/j.jalz.2015.03.005 26086185

[B86] Li Q. Huang H. Liu G. Lam K. Rutberg J. Green M. S.. (2009). Gain-of-function mutation of Nav1.5 in atrial fibrillation enhances cellular excitability and lowers the threshold for action potential firing. Biochem. Biophys. Res. Commun. 380, 132–137. 10.1016/j.bbrc.2009.01.052 19167345

[B87] Locke D. (1998). Gap junctions in normal and neoplastic mammary gland. J. Pathol. 186, 343–349. 10.1002/(SICI)1096-9896(199812)186:4<343::AID-PATH189>3.0.CO;2-X 10209481

[B88] Loscalzo J. Kohane I. Barabasi A-L. (2007). Human disease classification in the postgenomic era: a complex systems approach to human pathobiology. Mol. Syst. Biol. 3:124. 10.1038/msb4100163 17625512 PMC1948102

[B89] Louis E. D. (2014). Re-thinking the biology of essential tremor: from models to morphology. Parkinsonism Relat. Disord. 20(Suppl 1), S88–S93. 10.1016/S1353-8020(13)70023-3 24262197

[B90] Magnus-Levy A. (1895). Ueber den respiratorischen Gaswechsel unter Einfluss de Thyroidea sowie unter verschiedenen pathologische Zustand. Berlin Klin. Wochschr. 32, 650–652.

[B91] McAninch E. A. Bianco A. C. (2016). The History and future of treatment of hypothyroidism. Ann. Intern. Med. 164, 50–56. 10.7326/M15-1799 26747302 PMC4980994

[B92] McBrian M. A. Behbahan I. S. Ferrari R. Su T. Huang W-T. Kurdistani S. K.. (2013). Histone acetylation regulates intracellular pH. Mol. Cell. 49, 310–321. 10.1016/j.molcel.2012.10.025 23201122 PMC3893119

[B93] McKee A. C. Stern R. A. Nowinski C. J. Stein T. D. Alvarez V. E. Daneshvar D. H.. (2013). The spectrum of disease in chronic traumatic encephalopathy. Brain J. Neurol. 136, 43–64. 10.1093/brain/aws307 23208308 PMC3624697

[B94] Meinzer M. Lindenberg R. Darkow R. Ulm L. Copland D. Flöel A. (2014). Transcranial direct current stimulation and simultaneous functional magnetic resonance imaging. J. Vis. Exp. 86:e51730 10.3791/51730 PMC4181345 24796646

[B95] Mertens J. Wang Q-W. Kim Y. Yu D. X. Pham S. Yang B.. (2015). Differential responses to lithium in hyperexcitable neurons from patients with bipolar disorder. Nature 527, 95–99. 10.1038/nature15526 26524527 PMC4742055

[B96] Mirzaali M. J. Bürki A. Schwiedrzik J. Zysset P. K. Wolfram U. (2015). Continuum damage interactions between tension and compression in osteonal bone. J. Mech. Behav. Biomed. Mater. 49, 355–369. 10.1016/j.jmbbm.2015.05.007 26093346

[B97] Modesto K. Sengupta P. P. (2014). Myocardial mechanics in cardiomyopathies. Prog. Cardiovasc. Dis. 57, 111–124. 10.1016/j.pcad.2014.03.003 25081406

[B98] Montón C. Torres A. (1998). Lung inflammatory response in pneumonia. Monaldi Arch. Chest Dis. Arch. Monaldi Mal. Torace 53, 56–63. 9632909

[B99] Moolenaar W. H. Boonstra J. P. T. van der Saag de Laat S. W. (1981). Sodium/proton exchange in mouse neuroblastoma cells. J. Biol. Chem. 256, 12883–12887. 7309738

[B100] Moreira P. I. Siedlak S. L. Wang X. Santos M. S. Oliveira C. R. Tabaton M.. (2007). Increased autophagic degradation of mitochondria in Alzheimer disease. Autophagy 3, 614–615. 10.4161/auto.4872 17786024

[B101] Moriyama I. M. Loy R. M. A. Robb-Smith H. T. Hoyert D. L. Rosenberg H. M. (2011). History of the Statistical Classification of Diseases and Causes of Death, U.S. Department of Health and Human Services; Centers for Disease Control and Prevention; National Center for Health Statistics.

[B102] Nahorski S. R. Rogers K. J. (1973). *In vivo* effects of amphetamine on metabolites and metabolic rate in brain. J. Neurochem. 21, 679–686. 10.1111/j.1471-4159.1973.tb06012.x 4742145

[B103] Narendra D. Tanaka A. Suen F.-D. Youle R. J. (2009). Parkin-induced mitophagy in the pathogenesis of Parkinson disease. Autophagy 5, 706–708. 10.4161/auto.5.5.8505 19377297

[B104] Neeland I. J. Drazner M. H. Berry J. D. Ayers C. R. deFilippi C. Seliger S. L.. (2013). Biomarkers of chronic cardiac injury and hemodynamic stress identify a malignant phenotype of left ventricular hypertrophy in the general population. J. Am. Coll. Cardiol. 61, 187–195. 10.1016/j.jacc.2012.10.012 23219305 PMC3547631

[B105] Neviaser A. Andarawis-Puri N. Flatow E. (2012). Basic mechanisms of tendon fatigue damage. J. Shoulder Elbow. Surg. 21, 158–163. 10.1016/j.jse.2011.11.014 22244058 PMC3749775

[B106] Ni Z. Chen R. (2015). Transcranial magnetic stimulation to understand pathophysiology and as potential treatment for neurodegenerative diseases. Transl. Neurodegener. 4:22. 10.1186/s40035-015-0045-x 26579223 PMC4647804

[B107] Nicholls D. G. Ferguson S. J. Ferguson S. (2002). Bioenergetics. 3rd edn, San Diego, CA: Academic Press.

[B108] Numata S. Itatani K. Kanda K. Doi K. Yamazaki S. Morimoto K.. (2016). Blood flow analysis of the aortic arch using computational fluid dynamics. Eur. J. Cardio Thorac. Surg. 49, 1578–1585. 10.1093/ejcts/ezv459 26792932

[B109] Orešković D. Klarica M. (2011). Development of hydrocephalus and classical hypothesis of cerebrospinal fluid hydrodynamics: facts and illusions. Prog. Neurobiol. 94, 238–258. 10.1016/j.pneurobio.2011.05.005 21641963

[B110] Pandolfino J. E. Kwiatek M. A. Ho K. Scherer J. R. Kahrilas P. J. (2010). Unique features of esophagogastric junction pressure topography in hiatus hernia patients with dysphagia. Surgery 147, 57–64. 10.1016/j.surg.2009.05.011 19744454 PMC2891431

[B111] Pasteur (1881). On the germ theory. Science 2, 420–422. 10.1126/science.os-2.63.420 17830637

[B112] Pethig R. Kell D. B. (1987). The passive electrical properties of biological systems: their significance in physiology, biophysics and biotechnology. Phys. Med. Biol. 32:933. 10.1088/0031-9155/32/8/001 3306721

[B113] Petrov A. G. (2002). Flexoelectricity of model and living membranes. Biochim. Biophys. Acta 1561, 1–25. 10.1016/S0304-4157(01)00007-7 11988178

[B114] Petrov A. G. (2006). Electricity and mechanics of biomembrane systems: flexoelectricity in living membranes. Anal. Chim. Acta 568, 70–83. 10.1016/j.aca.2006.01.108 17761248

[B115] Picano E. Pellikka P. A. (2016). Ultrasound of extravascular lung water: a new standard for pulmonary congestion. Eur. Heart J. 37, 2097–2104. 10.1093/eurheartj/ehw164 27174289 PMC4946750

[B116] Plassman B. L. Havlik R. J. Steffens D. C. Helms M. J. Newman T. N. Drosdick D.. (2000). Documented head injury in early adulthood and risk of Alzheimer's disease and other dementias. Neurology 55, 1158–1166. 10.1212/WNL.55.8.1158 11071494

[B117] Pokorný J. Pokorný J. Kobilková J. Jandová A. Vrba J. Vrba J. (2014). Targeting mitochondria for cancer treatment - two types of mitochondrial dysfunction. Prague Med. Rep. 115, 104–119. 10.14712/23362936.2014.41 25626329

[B118] Porporato P. E. Dhup S. Dadhich R. K. Copetti T. Sonveaux P. (2011). Anticancer targets in the glycolytic metabolism of tumors: a comprehensive review. Front. Pharmacol. 2:49. 10.3389/fphar.2011.00049 21904528 PMC3161244

[B119] Porter C. Herndon D. N. Børsheim E. Chao T. Reidy P. T. Borack M. S.. (2014). Uncoupled skeletal muscle mitochondria contribute to hypermetabolism in severely burned adults. Am. J. Physiol. Endocrinol. Metab. 307, E462–E467. 10.1152/ajpendo.00206.2014 25074988 PMC4154069

[B120] Preston D. C. Shapiro B. E. (2012). Electromyography and Neuromuscular Disorders: Clinical-Electrophysiologic Correlations (Expert Consult - Online), Shanghai: Elsevier Health Sciences 2012.

[B121] Pupillo E. Messina P. Logroscino G. Zoccolella S. Chiò A. Calvo A. (2012). EURALS consortium, trauma and amyotrophic lateral sclerosis: a case-control study from a population-based registry. Eur. J. Neurol. Soc. 19, 1509–1517. 10.1111/j.1468-1331.2012.03723.x 22537412

[B122] Puwanant S. Park M. Popović Z. B. W. Tang H. W. Farha S. Thomas J. D.. (2010). Ventricular geometry, strain, and rotational mechanics in pulmonary hypertension. Circulation 121, 259–266. 10.1161/CIRCULATIONAHA.108.844340 20048214 PMC2846516

[B123] Ratheiser K. M. Brillon D. J. Campbell R. G. Matthews D. E. (1998). Epinephrine produces a prolonged elevation in metabolic rate in humans. Am. J. Clin. Nutr. 68, 1046–1052. 10.1093/ajcn/68.5.1046 9808221

[B124] Reynolds E. (2001). Todd, Hughlings Jackson, and the electrical basis of epilepsy. Lancet Lond. Engl. 358, 575–577. 10.1016/S0140-6736(01)05710-5 11520547

[B125] Robicsek F. Thubrikar M. J. (2002). Mechanical stress as cause of aortic valve disease. Presentation of a new aortic root prosthesis. Acta Chir. Belg. 102, 1–6. 10.1080/00015458.2002.11679253 11925731

[B126] Rumsey W. L. Pawlowski M. Lejavardi N. Wilson D. F. (1994). Oxygen pressure distribution in the heart *in vivo* and evaluation of the ischemic “border zone”. Am. J. Physiol. 266, H1676–H1680. 8184947 10.1152/ajpheart.1994.266.4.H1676

[B127] Runyon B. A. (1994). Malignancy-related ascites and ascitic fluid “humoral tests of malignancy. J. Clin. Gastroenterol. 18, 94–98. 10.1097/00004836-199403000-00002 8189030

[B128] Safar M. E. Levy B. I. Struijker-Boudier H. (2003). Current perspectives on arterial stiffness and pulse pressure in hypertension and cardiovascular diseases. Circulation 107, 2864–2869. 10.1161/01.CIR.0000069826.36125.B4 12796414

[B129] Safar M. E. Nilsson P. M. Blacher J. Mimran A. (2012). Pulse pressure, arterial stiffness, and end-organ damage. Curr. Hypertens. Rep. 14, 339–344. 10.1007/s11906-012-0272-9 22555981

[B130] Saji N. Toba K. Sakurai T. (2016). Cerebral small vessel disease and arterial stiffness: tsunami effect in the brain? Pulse Basel Switz, 3, 182–189. 10.1159/000443614 27195239 PMC4865071

[B131] Sardet C. Franchi A. Pouysségur J. (1989). Molecular cloning, primary structure, and expression of the human growth factor-activatable Na+/H+ antiporter. Cell 56, 271–280. 10.1016/0092-8674(89)90901-X 2536298

[B132] Schilli R. Breuer R. I. Klein F. Dunn K. Gnaedinger A. Bernstein J.. (1982). Comparison of the composition of faecal fluid in Crohn's disease and ulcerative colitis. Gut 23, 326–332. 10.1136/gut.23.4.326 7076010 PMC1419741

[B133] Schmidt M. L. Zhukareva V. Newell K. L. Lee V. M. Trojanowski J. Q. (2001). Tau isoform profile and phosphorylation state in dementia pugilistica recapitulate Alzheimer's disease. Acta Neuropathol. 101, 518–524. 10.1007/s004010000330 11484824

[B134] Schwartz L. (2004). Cancer - Between Glycolysis and Physical Constraint: Between Glycolysis and Physical Constraint. Springer Science & Business Media.

[B135] Schwartz L. (2014). Is liver disease caused by increased pressure? interstitial pressure as a causative mechanism in carcinogenesis and in the differential blood supply in liver tumors from the hepatic artery. J. Liver. 3:156 10.4172/2167-0889.1000156

[B136] Schwartz L. Abolhassani M. Guais A. Sanders E. J. Steyaert M. Israël M.. (2010). A combination of α lipoic acid and calcium hydroxycitrate is efficient against mouse cancer models: preliminary results. Oncol. Rep. 23, 1407–1416. 10.3892/or_00000778 20372858

[B137] Schwartz L. Abolhassani M. Pooya M. Steyaert J-M. Wertz X. Chaumet-Riffaud P.. (2008). Hyperosmotic stress contributes to mouse colonic inflammation through the methylation of protein phosphatase 2A. Am. J. Physiol. Gastrointest. Liver Physiol. 295, G934–G941. 10.1152/ajpgi.90296.2008 18755808

[B138] Schwartz L. Balosso J. Baillet F. Brun B. Amman J. P. Sasco A. J. (2002). Cancer: the role of extracellular disease. Med. Hypotheses 58, 340–346. 10.1054/mehy.2001.1539 12027530

[B139] Schwartz L. Guais A. Pooya M. Abolhassani M. (2009). Is inflammation a consequence of extracellular hyperosmolarity? J. Inflamm. Lond. Engl. 6:21. 10.1186/1476-9255-6-21 19549308 PMC2709204

[B140] Schwartz L. Supuran C. T. Alfarouk K. O. (2017). The Warburg effect and the hallmarks of cancer. Anti Cancer Agents Med. Chem 17, 164–170. 10.2174/1871520616666161031143301 27804847

[B141] Seyfried T. N. Shelton L. M. (2010). Cancer as a metabolic disease. Nutr. Metab. 7:7. 10.1186/1743-7075-7-7 20181022 PMC2845135

[B142] Sheean G. L. (2012). Quantification of motor unit action potential energy, Clin. Neurophysiol. 123, 621–625. 10.1016/j.clinph.2011.08.009 21903464

[B143] Shoham N. Gefen A. (2012). Deformations, mechanical strains and stresses across the different hierarchical scales in weight-bearing soft tissues. J. Tissue Viability 21, 39–46. 10.1016/j.jtv.2012.03.001 22520396

[B144] Silver F. H. Bradica G. (2002). Mechanobiology of cartilage: how do internal and external stresses affect mechanochemical transduction and elastic energy storage? Biomech. Model. Mechanobiol. 1, 219–238. 10.1007/s10237-002-0017-9 14586701

[B145] Singhal A. Parker S. Linsell L. Serjeant G. (2002). Energy intake and resting metabolic rate in preschool Jamaican children with homozygous sickle cell disease. Am. J. Clin. Nutr. 75, 1093–1097. 10.1093/ajcn/75.6.1093 12036818

[B146] Sipe J. D. (1995). Acute-phase proteins in osteoarthritis. Semin. Arthritis Rheum. 25, 75–86. 10.1016/S0049-0172(95)80020-4 8578314

[B147] Song W. Xiao Y. Chen H. Ashpole N. M. Piekarz A. D. Ma P.. (2012). The human Nav1.5 F1486 deletion associated with long QT syndrome leads to impaired sodium channel inactivation and reduced lidocaine sensitivity. J. Physiol. 590, 5123–5139. 10.1113/jphysiol.2012.235374 22826127 PMC3497567

[B148] Srivastava A. Mannam P. (2015). Warburg revisited: lessons for innate immunity and sepsis. Front. Physiol. 6:70. 10.3389/fphys.2015.00070 25806001 PMC4353299

[B149] Staples J. F. (2016). Metabolic flexibility: hibernation, torpor, and estivation. Compr. Physiol. 6, 737–771. 10.1002/cphy.c140064 27065167

[B150] Stein T. D. Alvarez V. E. McKee A. C. (2014). Chronic traumatic encephalopathy: a spectrum of neuropathological changes following repetitive brain trauma in athletes and military personnel. Alzheimers Res. Ther. 6:4. 10.1186/alzrt234 24423082 PMC3979082

[B151] Streitberger J.-K. Wiener E. Hoffmann J. Freimann F. B. Klatt D. Braun J.. (2011). *In vivo* viscoelastic properties of the brain in normal pressure hydrocephalus. NMR Biomed. 24, 385–392. 10.1002/nbm.1602 20931563

[B152] Stuart G. Spruston N. Häusser M. (2016). Dendrites. Oxford: Oxford University Press.

[B153] Sun P. Qin J. Campbell K. (2015). Fatigue modeling via mammalian auditory system for prediction of noise induced hearing loss. Comput. Math. Methods Med. 2015:753864. 10.1155/2015/753864 26691685 PMC4672119

[B154] Swenson E. R. Maggiorini M. Mongovin S. J. Gibbs S. R. Greve I. Bärtsch P. (2002). Pathogenesis of high-altitude pulmonary edema: inflammation is not an etiologic factor. JAMA 287, 2228–2235. 10.1001/jama.287.17.2228 11980523

[B155] Szturmowicz M. Tomkowski W. Fijalkowska A. Burakowski J. Sakowicz A. Filipecki S. (1997). The role of carcinoembryonic antigen (CEA) and neuron-specific enolase (NSE) evaluation in pericardial fluid for the recognition of malignant pericarditis. Int. J. Biol. Markers 12, 96–101. 9479590 10.1177/172460089701200302

[B156] Thakral G. Lafontaine J. Najafi B. Talal T. K. Kim P. Lavery L. A. (2013). Electrical stimulation to accelerate wound healing. Diabet. Foot Ankle. 4. 10.3402/dfa.v4i0.22081 24049559 PMC3776323

[B157] Theuvenet P. J. Dunajski Z. Peters M. J. van Ree J. M. (1999). Responses to median and tibial nerve stimulation in patients with chronic neuropathic pain, Brain Topogr. 11, 305–313. 10.1023/A:1022210704505 10449261

[B158] Thompson D. W. (1942). On Growth and Form. Cambridge: Cambridge University Press.

[B159] Uryu K. Chen X-H. Martinez D. Browne K. D. Johnson V. E. Smith D. H.. (2007). Multiple proteins implicated in neurodegenerative diseases accumulate in axons after brain trauma in humans. Exp. Neurol. 208, 185–192. 10.1016/j.expneurol.2007.06.018 17826768 PMC3979356

[B160] Valdez M. Balachandran B. (2013). Longitudinal nonlinear wave propagation through soft tissue. J. Mech. Behav. Biomed. Mater. 20, 192–208. 10.1016/j.jmbbm.2013.01.002 23510921

[B161] van Alphen H. A. (1986). Migraine, a result of increased CSF pressure: a new pathophysiological concept (preliminary report). Neurosurg. Rev. 9, 121–124. 10.1007/BF01743062 3736895

[B162] Villafuerte F. C. Corante N. (2016). Chronic mountain sickness: clinical aspects, etiology, management, and treatment. High Alt. Med. Biol. 17, 61–69. 10.1089/ham.2016.0031 27218284 PMC4913504

[B163] Vincent K. R. Conrad B. P. Fregly B. J. Vincent H. K. (2012). The pathophysiology of osteoarthritis: a mechanical perspective on the knee joint, PM R. 4, S3–S9. 10.1016/j.pmrj.2012.01.020 22632700 PMC3635670

[B164] Visser A. W. de Mutsert R. le Cessie S. den Heijer M. Rosendaal F. R. Kloppenburg M. (2014). The relative contribution of mechanical stress and systemic processes in different types of osteoarthritis: the NEO study. Ann. Rheum. Dis. 74, 1842–1877. 10.1136/annrheumdis-2014-eular.3179 24845389

[B165] Warburg O. (1956). On the origin of cancer cells. Science 123, 309–314. 10.1126/science.123.3191.309 13298683

[B166] Wiener T. C. (2012). Space obstructive syndrome: intracranial hypertension, intraocular pressure, and papilledema in space. Aviat. Space Environ. Med. 83, 64–66. 10.3357/ASEM.3083.2012 22272520

[B167] Wilson M. H. Imray H. E. Hargens A. R. (2011). The headache of high altitude and microgravity–similarities with clinical syndromes of cerebral venous hypertension. High Alt. Med. Biol. 12, 379–386. 10.1089/ham.2011.1026 22087727

[B168] Yakushev I. Gerhard A. Müller M. J. Lorscheider M. Buchholz G.-H. Schermuly I.. (2011). Relationships between hippocampal microstructure, metabolism, and function in early Alzheimer's disease. Brain Struct. Funct. 216, 219–226. 10.1007/s00429-011-0302-4 21318476

[B169] Zetterberg A. Engström W. (1981). Mitogenic effect of alkaline pH on quiescent, serum-starved cells. Proc. Natl. Acad. Sci. U.S.A. 78, 4334–4338. 10.1073/pnas.78.7.4334 6945587 PMC319784

[B170] Zhang P. C. Keleshian A. M. Sachs F. (2001). Voltage-induced membrane movement. Nature 413, 428–432. 10.1038/35096578 11574890

[B171] Zhang Z. Tang S. Zhu L. Wu G. Jiang Z. Shi B. (2008). [Stresses in portal venous system of pre-hepatic portal hypertension (PHT) rabbits], Sheng Wu Yi Xue Gong Cheng Xue Za Zhi. J. Biomed. Eng. Shengwu Yixue Gongchengxue Zazhi. 25, 1322–1326. 19166202

